# Polarization Insensitive, Wide-Angle, Ultra-wideband, Flexible, Resistively Loaded, Electromagnetic Metamaterial Absorber using Conventional Inkjet-Printing Technology

**DOI:** 10.1038/s41598-019-48761-6

**Published:** 2019-08-26

**Authors:** Stylianos D. Assimonis, Vincent Fusco

**Affiliations:** School of Electronics, Electrical Engineering and Computer Science, Queen’s University Belfast, Belfast, BT3 9DT United Kingdom

**Keywords:** Electrical and electronic engineering, Metamaterials

## Abstract

A novel, polarization insensitive, wide-angle and broadband electromagnetic metamaterial absorber, which can cover either a flat or a bent geometry, is presented in this work. The periodic geometry has a unit-cell, which consists of four split ring resonators, which are sequentially rotated around the unit-cell axis progressively by 90 deg. First, the metallic parts of the geometry consists of low resistivity copper traces. Next, in order to increase the frequency bandwidth, resistively loaded traces, printed by conventional inkjet printer are used to replace the copper ones. For normal incidence, simulated and measurement results shown that the proposed flat absorber exhibits absorption efficiency higher than 0.8 for 6.9–29.9 GHz (i.e., bandwidth of 125%) regardless of polarization, while the curved absorber for 6.6–29 GHz (i.e., bandwidth of 126%) or for 10.5–29.6 GHz (i.e., bandwidth of 95%), depending the polarization. For oblique incidence and for TE or TM polarized incident wave it presents bandwidth of 118% (7.7–29.9 GHz) or 100.5% (9.9–29.9 GHz), respectively, for an incident angular range of 0–45 deg. Finally, the proposed absorber has thickness of 3.89 mm, corresponding to *λ*/11.7 at its lowest operation frequency.

## Introduction

Metamaterials are inherently inhomogeneous structures that usually consist of periodically repeated unit cells. Their properties mainly arise from their geometric details rather than their constituting material properties. A particular type of metamaterial, the metamaterial perfect absorber, has attracted significant attention due to its ability to offer near unity absorption of electromagnetic waves. The first perfect metamaterial absorber was presented by N. I. Landy *et al*. in 2008^1^. Since then, the scientific effort was focused on the design of polarization insensitive and wide-angle^2–8^, tunable^9–11^, flexible^12^, multi-band^13–21^, and wideband electromagnetic absorbers by using 3-dimensional (3D) structures^22–24^, copper traces and chip resistors^25–30^, or resistively loaded patters^31–37^ This work is focused on the latter group since usually this group of absorbers exhibit superior performance in terms of bandwidth: it will be shown that, the absorption mechanism in these structures is because of the losses in the resistive parts rather in the dielectrics and that, in turn, leads to wider absorption frequency bandwidth.

Specifically, in^23^ the presented electromagnetic absorber, which consists of a conductive plastic disk, was fabricated through 3D-printing technology and lies between two substrates (one of the later is grounded), presents simulated absorbance for normal incidence higher than 0.9 for 16.3–54.3 GHz, while measured results reveals operation from 23.3 GHz. Similarly, in^24^ the authors fabricated via 3D-printing technology and tested via measurements a 3D-resistive absorber, which is much like a honeycomb of resistive walls. The geometry presents absorbance bandwidth of 148.7% and is robust for oblique incidence of a plane wave with transverse magnetic (TM) polarization. However, both the above electromagnetic absorbers^23,24^ are relatively thick (around ~*λ*_min_/6.1 at their lowest operation frequency) and demanding in terms of fabrication process.

In^28^ the proposed geometry consists of chip resistors, soldered onto a copper pattern, which lies in between two substrates (one is grounded). For normal incidence the absorber exhibits a fractional bandwidth (FBW) of 100%, while for oblique incidence it presents FBW from 77.3% to 90.5% up to 60 deg, depending the polarization of the incident plane wave. The absorber in^29^ consists of two patterned layers (one is scaled to the other) and chip resistors, as well, but exhibits wider FBW (112%) than^28^ for normal incidence: in general, the use of multiple pattern layers leads to wider absorbance, since each of these, resonates at a different frequency, and thus, the final absorbance consists of multiple maxima, which produce a wide bandwidth^38^. A similar technique is used by the authors in^30^: now the scaled pattern and the original one lie on the same layer, resulting FBW = 126.8%.

In order to reduce the fabrication effort but to keep the FBW as wide as it is possible, the chip resistors, which need soldering during the fabrication process, are replaced by resistively loaded patterns: now the captured RF energy dissipates in the latter and not in the chip resistors, as it was previously. In^35^ a restively loaded electromagnetic absorber was presented: the structure is thin (thickness of only *λ*_min_/12.3) and for normal incidence has FBW = 102%. However, the absorber is weak in terms of absorbance bandwidth for oblique incidence since its FBW is reduced to 54% and 50%, for TE- and TM-polarization, up to 45 deg angle of incidence. In^36^, three scaled resistive patterns are used in a multilayer geometry, which remains relatively thin (*λ*_min_/11) and the FBW is increased to 132.9% for normal incidence, as expected.

The contribution of this work is the presentation of a systematic, versatile and low complexity method for the design of a type of polarization insensitive and wide-angle electromagnetic metamaterial absorber, wherein the basic idea is the form of super-cells with 90 rotational degree symmetry. In this work the proposed method was applied to a squared split ring resonator (SSRR) structure, but it could be applied to any resonator. The resulting absorber is shown to be an electrically thin (thickness of *λ*_min_/11.7), ultra-wideband, polarization insensitive having wide-angle absorption. The structure is easily fabricated and low-cost, since it was printed using a conventional inkjet printer deploying conductive ink, consisting of silver nano-particles diluted in aqua^39^. Additionally, the proposed absorber is flexible: it managed to reduce the radar cross section (RCS) of a metallic pole of radius about *λ*_min_/7 by an average of 10 dB for a frequency zone of about 30 GHz, depending the polarization of the incident field. Based on the literature, the proposed structure is the first flexible, ultra-wideband, polarization insensitive, wide-angle, easily fabricated through conventional inkjet printing techniques, electrically thin electromagnetic absorber (Table 1).

**Table 1:** Comparison of Ultra-Wideband Electromagnetic Absorbers.

work	FBW % (Frequency in GHz)					Thickness in *λ*_min_	Pattern layers	Fabrication technique
	Flat			Curved				
	Normal	Oblique		Normal				
	TE or TM	TE	TM	E∥	H∥			
^23^^−,*^	107.6 (16.3–54.3)	N/A		N/A		1/6.8	1	3D printed conductive plastic
^24^^+,*^	148.7 (3.53–24)	N/A	148.5 (3.53–24) *θ* ≤ 70°	N/A		1/5.5	1	3D printed honeycomb with resistive walls
^28^^+,*^	100 (4.8–14.4)	77.3 (4.6–10.4) *θ* ≤ 40°	90.5 (4.6–12.2) *θ* ≤ 60°	N/A		1/12	2	soldering
^29^^+,*^	112 (5.1–18.08)	N/A		NA		1/12.8	2	soldering
^30^^+,*^	126.8 (N/A)	N/A		N/A		1/11.4	1	soldering
^32^^+,**^	85.7 (15.6–39)	90.9 (15–40) *θ* ≤ 30°	80 (15–35) *θ* ≤ 30°	N/A		1/10	1	indium tin oxide
^35^^+,*^	102 (7.8–24)	54 (N/A) *θ* ≤ 45°	50 (N/A) *θ* ≤ 45°	N/A		1/12.3	1	silk printing technique
^36^^+,*^	132.9 (7.2–35.7)	N/A		N/A		1/11	3	silk printing technique
This work^+,**^	125 (6.9–29.9)	118 (7.7–29.9) *θ* ≤ 45°	100.5 (9.9–29.9) *θ* ≤ 45°	126 (6.6–29)	95 (10.5–29.6)	1/11.7	1	printed via conventional inkjet printer

^+^measurement, ^−^simulation, ^*^A > 0.9, ^**^A > 0.8.

Only numeric data provided by the authors are tabulated.

## Results and Discussion

This section presents performance in terms of absorptivity of two types metamaterial absorbers: first (from now on called *copper-absorber*), the metallic parts of the periodic geometry are made of copper, while second (from now on, called *printed-absorber*), are inkjet-printed resistively loaded traces. In the both cases, the unit-cell of the periodic geometry consists of four SSRRs, which are co-planar and are sequentially rotated by an angle of 90 deg. around the central, unit-cell axis.

Because of their rotational symmetry, both absorbers present polarization insensitivity, while because the unit-cell’s dimension, which is electrically small (*λ*/11.3 at resonance frequency and *λ*_min_/6.9 for the lower absorption frequency for the copper- and printed-absorber, respectively), they are wide-angle. The main difference between the two absorber types is the absorption frequency bandwidth: the copper-absorbers are narrow-band, with fractional bandwidth (FBW) of 80% absorbance on the order of 2%, while the resistively loaded absorbers present FBW of the order of 100%^31–37^ for 80% absorbance. The reason for this is that, in the copper-absorber the captured energy mainly dissipates in the substrate rather than in the copper traces, while, in the printed-absorber, energy mainly dissipates in the resistively loaded traces, which removing the resonant nature of the SSRRs by re-distributing their currents. Based on the latter, the absorption mechanism changes, resulting in wideband operation, as will be explained.

### Copper Absorber

**Figure 1:**
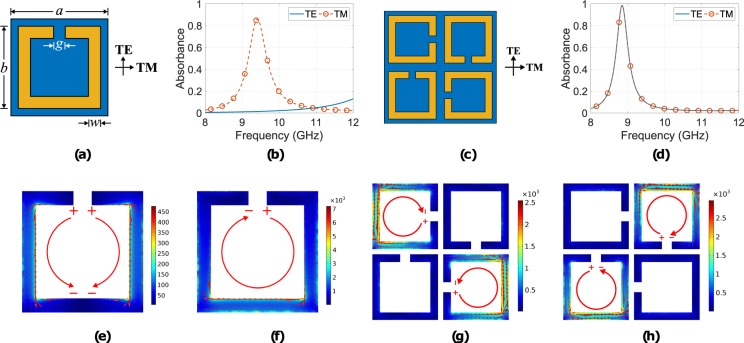
The split ring resonator unit-cell using low resistivity copper (5.8 × 10^7^ S/m) with dimensions *a* = 3 mm (*λ*/11.3 at resonance frequency GHz), *d* = 2.8 mm, *w* = 0.32 mm and *g* = 0.42 mm (**a**) and the simulated absorbance for normal incidence and for TE or TM polarization (**b**): only for the TM polarization does the geometry act as an absorber. Also depicted is the super-cell (**c**), consisting of four of the basis unit-cells, which are sequentially rotated by 90 deg and the simulated *A* for the latter structure, for normal incidence and for TE or TM polarization (**d**): identical *A* for both polarizations. The surface current distribution for the unit-cell, for TE (**e**) and TM (**f**) polarization and for the super-cell for TE (**g**) and TM (**h**) polarization, simulated at the corresponding resonance frequencies and for normal incidence, is also depicted: it is observed that the currents, which form loops are dominant in terms of their intensity, and these lead to absorption.

The geometry of a typical SSRR is presented in Fig. 1a: the unit-cell has dimension of 3 mm with copper ring of conductivity 5.8 × 10^7^ S/m, size, width and gap of 2.8, 0.32 and 0.42 mm, respectively, lying on an FR-4 grounded substrate with thickness of 0.8 mm, dielectric permittivity of *ε*_*r*_ = 4 and losses of tan*δ* = 0.02. A periodic geometry is formed by applying periodic boundary conditions around the unit-cell and the reflection coefficient for normal incidence and for both polarizations transverse electric (TE) and magnetic (TM) behaviour is estimated through electromagnetic simulation using the commercial solver COMSOL Multiphysics^40^: the electric field component is also depicted in the Fig. 1a. For an absorber periodic geometry, absorbance is given by,

1 $$A=1-{|{\rm{\Gamma }}|}^{2}-{|{\rm{T}}|}^{2},$$

where, Γ and T is the reflection and transmission coefficient, respectively, while since the geometry is grounded, thus T → 0, is

2 $$A=1-{|{\rm{\Gamma }}|}^{2}.$$

Figure 1b depicts *A* for both polarization cases at normal incidence of the single SSRR (Fig. 1a): it is evident that only for the TM case does the geometry act as an absorber, i.e., it presents *A* higher than 80%, at 9.4 GHz. Next a *super-cell* is formed by using four of the basic unit-cells, which are sequentially rotated by 90 deg. and the final geometry is depicted in Fig. 1c). *A* is simulated for normal incidence and for both TE, TM polarization, and the results are depicted in Fig. 1d: *A* is identical for the both TE-, TM-cases, as expected because of the unit-cell’s symmetry, presenting maximum of 98.5% at 8.84 GHz and has FBW = 2.1%. The super-cell’s size is *λ*/5.65 at the resonance frequency, and thus, it remains electrically compact.

**Figure 2:**
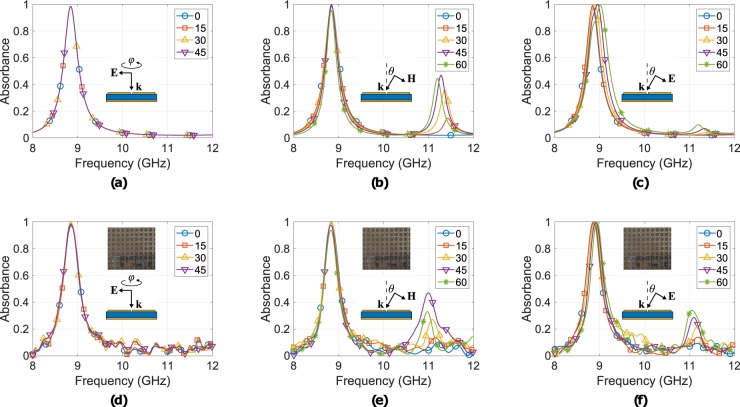
Simulated (**a**–**c**) versus measured (**d**–**f**) results for the copper-absorber absorbance for normal incidence as angle *ϕ* varies form 0 deg. to 45 deg. (**a**,**d**) and for oblique incidence as *θ* varies from 0 deg. to 60 deg. for TE- (**b**,**e**) and TM-polarization (**c**,**f**): here good agreement is observed. Also depicted is the fabricated and measured geometry as inset.

In order to investigate the absorbance mechanism, the surface current distribution was simulated, for both the unit and the super-cell at the resonance frequency. Figure 1e,f depicts the results for TE and TM polarization, respectively, for normal incidence and for the unit-cell (Fig. 1a) at 9.4 GHz: it is observed that for the TE-case reverse currents (red lines) are induced, resulting in no absorption, while for the TM-case the current forms a loop, which runs along the whole SSRR periphery and results in absorption. Moreover, for the TE-case, the maximum current magnitude (≈450 A/m) is much smaller than the corresponding current at the TM-case (≈7 × 10^3^ A/m). Similarly, Fig. 1g,h depict the surface current distribution, but now for the super-cell (Fig. 1c) at 8.84 GHz. For the TE-case, (Fig. 1g), the dominant SSRRs in terms of interaction with the incident wave is the upper-left and the bottom-right, as expected, while for the TM-case (Fig. 1h) the top-right and bottom-left are active. Hence, at each polarization (TE or TM), the super-cell absorbs energy since two of its four sub unit-cells resonate, and thus, the super-cell presents polarization insensitivity: the latter is evident in Fig. 2a, which depicts the simulated absorbance for normal incidence as the electric (or magnetic, as well) field component of the incident plane wave steers from 0 to 45 deg. (angles greater than 45 deg. were not tested since the super-cell presents 45 deg. symmetry). Thus, for normal incidence, and based on the surface current distribution, the absorption mechanism is due to the interaction of the electric field of the plane wave with the SSRRs. Specifically, the latter are coupled together and that causes strong resonance driven surface current loops, which in turns produces ohmic losses because of the finite conductivity of the copper and due to their interaction with the lossy substrate (it will be explained later how the energy dissipates). For this case, there is no normal magnetic field component on the SSRRs, hence, surface currents are not induced because of the latter, and thus, magnetic field does not contribute to the absorption mechanism.

Next, polarisation insensitivity was tested for oblique incidence. Figure 2b depicts the results for TE polarisation. As angle of incidence increases from 0 to 60 deg., absorbance remains higher than 96% at 8.84 GHz. However, a second maxima occurs at around 11.5 GHz. For this case, there is a magnetic field component normal to the SSRRs, and thus, surface loop currents are induced, which in turn, contribute to the absorption mechanism, as explained before. The second maxima observed in the absorbance is because of these currents: as the angle of incidence *θ* increases, the normal magnetic field component magnitude becomes higher, which in turn increases the induced surface loop currents, which lead to higher losses and thus, to higher absorbance. For *θ* > 45 deg., the latter phenomenon saturates, and there is no further increment for the absorbance’s second maxima. On the other hand, the first maxima at 8.84 GHz remains almost the same as *θ* increases, or equivalently as the normal magnetic field component increases, This is a result of the coupling between the tangential electric field of the plane wave and the SSRRs, which remains constant as *θ* increases.

Figure 2c again depicts the absorbance for oblique incidence but now for TM polarisation. It is clear that *A* always remains higher than 98.5%, as it was for the normal incidence case, but now there is a slight shift of the maxima to higher frequencies. The latter results from the tangential to the SSRRs electric field component of the incident plane wave is changing as *θ* increases: now, as the incident plane wave impinges on the SSRRs the apparent equivalent SSRR’s electrical size changes from the perspective of the incident signal: the inducted currents by the tangential electric field form loops with smaller equivalent electrical length, and thus, the resonance frequency increases.

Figure 2 also depicts the corresponding measurements for normal and oblique incidence, for both polarizations: here good agreement is observed. Also depicted is the fabricated (though chemical etching process) and measured geometry as inset. The measurement procedure and set-up will be explained in the Methods section. It is noted that time domain gating and smoothing techniques (i.e., moving average filter)^41^ were applied over the measurement’s data.

### Salisbury Screen Absorber

A typical resistively loaded absorber is the Salisbury screen (SC) geometry, which is used for reflection reduction of incident radio waves to a surface. Proposed by Salisbury^42^ in the early of 1950s it usually consists of three layers: a screen, which corresponds to a specific equivalent electric surface resistance *Z*_S_, a loss-less dielectric substrate of thickness *λ*/4, where *λ* is the wavelength of the absorbed radio wave, and a ground plane: the wave initially impinges on the surface and part of it is reflected, while the other part propagates into the substrate until it is reflected from the ground plane. At the ground plane, the first and the second reflected parts are out of phase (i.e., phase difference of 180 deg.), resulting in reflection suppression.

**Figure 3:**
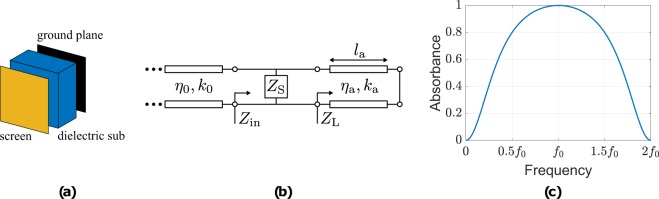
The Salisbury screen geometry (**a**), the equivalent circuit schematic (**b**) and the frequency response in terms of absorbance: the structure operates (i.e., *A* ≥ 0.8) from 0.5*f*_0_ to 1.5*f*_0_, i.e., it presents a bandwidth of 100%.

The Salisbury screen geometry and its equivalent circuit schematic are depicted in Fig. 3, where *η*_a_, *k*_a_ = 2*π*/*λ* and *l*_a_ is the characteristic impedance, wavenumber and thickness of the substrate, respectively. Based on transmission line theory (TLT) and since the transmission line is terminated in a short-circuit through a substrate of *λ*/4–thickness (i.e., *l*_a_ = *λ*/4) is

3 $${Z}_{{\rm{L}}}=j{\eta }_{{\rm{a}}}\,\tan ({k}_{{\rm{a}}}{l}_{{\rm{a}}})\to \infty ,$$

thus, the input impedance of the geometry is given by,

4 $${Z}_{{\rm{in}}}=\frac{{Z}_{{\rm{S}}}{Z}_{{\rm{L}}}}{{Z}_{{\rm{S}}}+{Z}_{{\rm{L}}}}\to {Z}_{{\rm{S}}}.$$

For perfect absorption, the structure should be impedance matched to the vacuum, and hence,

5 $${Z}_{{\rm{in}}}={\eta }_{0}^{\ast }={\eta }_{0}$$

where ${\eta }_{0}=\sqrt{{\mu }_{0}/{\varepsilon }_{0}}\approx 120\pi$ ${\rm{\Omega }}$ is the wave impedance of the vacuum and {*} denotes conjugated match. Based on (4), (5)

6 $${Z}_{{\rm{S}}}\to {\eta }_{0}.$$

Thus, in a typical Salisbury absorber the screen is represented by a real resistance, i.e., *Z*_s_ = *R*, i.e. pure resistance.

Figure 3c shows the estimated frequency response of a Salisbury screen. The latter was designed to absorb at *f*_0_ frequency and operates (i.e., *A* ≥ 0.8) from 0.5*f*_0_ to 1.5*f*_0_, and thus, it has bandwidth of 100%. The above analysis has revealed that, by using resistively loaded surfaces it is possible to design a wideband absorber, which has thickness of at least *λ*/4. For thinner geometries with ever wider bandwidth, the above analysis leads *Z*_s_ to be a complex impedance and its circuit equivalent is an RLC circuit^43,44^, as will be explained.

### Printed Absorber

**Figure 4:**
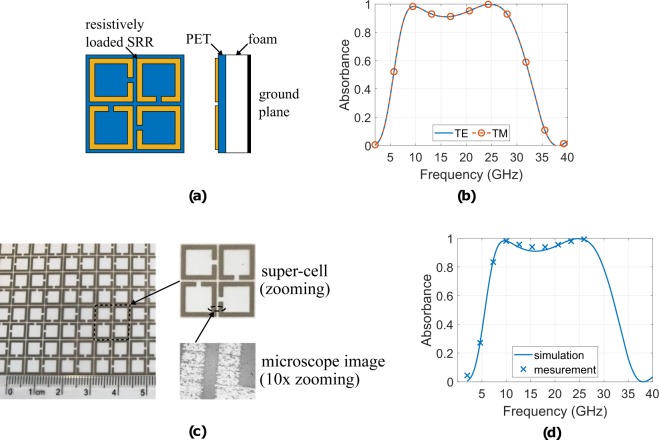
The resistive inked printed absorber geometry (**a**) and the simulated absorbance (**b**) for normal incidence and for both polarizations, i.e., TE and TM: the proposed absorber is wideband and operates from 6.9 to 29.9 GHz, i.e., it presents FBW = 125%. It is also depicted the fabricated geometry (**c**) and the measurement versus simulation results for normal incidence (**d**): a very good agreement is observed.

In order to design a polarization insensitive, wide angle and wideband absorber, the rotated structure presented in the previous section, but now with resistively loaded traces has been used. The proposed geometry is depicted in Fig. 4. The super-cell (unit-cell) has now size of 12.59 mm (6.3 mm) with resistively loaded rings of 27 Ω/sq, size, width and gap of 5.84, 0.68 and 0.88 mm, respectively, while they lie on a substrate with a permittivity of *ε*r = 2.5, losses of tan*δ* = 0.0009 and thickness of *h*_1_ = 0.14 mm. The latter is mounted on a grounded foam-substrate, with *ε*_r_ = 1.05, zero losses (tan*δ* = 0) and thickness of *h*_2_ = 3.75 mm. Thus, the absorber has thickness of *h*_1_ + *h*_2_ = 3.89 mm. The geometry was studied through full electromagnetic analysis is terms of absorbance. Specifically, periodic boundary conditions were applied again around the super-cell and the reflection coefficient of plane wave for normal incidence for both polarizations, TE and TM, was estimated using the COMSOL Multiphysics solver. Figure 4b depicts the results: absorber operates (*A* ≥ 0.8) for 6.9–29.9 GHz, i.e., has FBW = 125% for both polarizations. For the lower frequency, the super-cell has thickness of *λ*_min_/11.17, and hence, is much thinner than the Salisbury screen, while presenting wider bandwidth, as well. Additionally, two maxima of absorbance are observed, i.e., 0.985 and 0.999 at 9.7 and 24.7 GHz, respectively.

Next the absorber was fabricated and measured. The resistively loaded SSRRs were printed through the conventional inkjet printer Epson Stylus C88 + on a coated polyethylene terephthalate (PET) sheet (*ε*_*r*_ = 2.5, tan*δ* = 0.0009 of thickness 0.14 mm). Between the ground plane (i.e., metallic plate of copper) and the PET-sheet the foam-substrate (*ε*_*r*_ = 1.05) of thickness 3.75 mm was placed. In order to achieve resistively loaded SSRRs, the electrically conductive ink Metalon^TM^ JS-B25P^45^ was used. The latter was diluted with water as the solvent, stored in an empty inkjet cartridge and used by the printer. The surface impedance in this work was fixed at *R*_s_ = 27 Ω/sq and it was adjusted by two ways: first by forming specific volume ratio of the conductive ink to water (i.e., 1:5) and second, by adjusting the grey scale (through the RGB-code) on the printer (i.e., RGB-code (29,29,29)), while the whole fabrication procedure will be explained in the Methods section and it is also well explained in^39^. The fabricated geometry is depicted in Fig. 4c, where the super-cell is presented, as well, enlarged. Figure 4d depicts the measured versus simulated results for the normal incidence: here good agreement is observed. It is noted that the printed absorber was tested for normal incidence only from 1 GHz to 26 GHz due to facilities limitations in our laboratory. Again, time domain gating and smoothing techniques (i.e., moving average filter)^41^ were applied over the measurement’s data.

It is known that the captured by the absorber power dissipates either on its dielectric or metallic parts. Specifically, for the dielectric parts the power losses can be obtained through^46^

7 $${P}_{d}=\pi \varepsilon f\,\tan \,\delta {{\iiint }_{{V}_{d}}|E|}^{2}{\rm{d}}V$$

where *V*_*d*_, *ε* and tan*δ* is the substrate’s volume, permittivity and tangential losses, respectively, *E* the electric field and *f* the frequency of the incident plane wave, while for the metallic parts^46^,

8 $${P}_{m}=\frac{1}{2}\sqrt{\frac{\pi \mu f}{\sigma }}{{\iint }_{{S}_{m}}|H|}^{2}{\rm{d}}S$$

where, *S*_*m*_, *μ* and *σ* is the conductor’s surface, permeability and conductivity, respectively and *H* the magnetic field of the incident plane wave.

**Figure 5:**
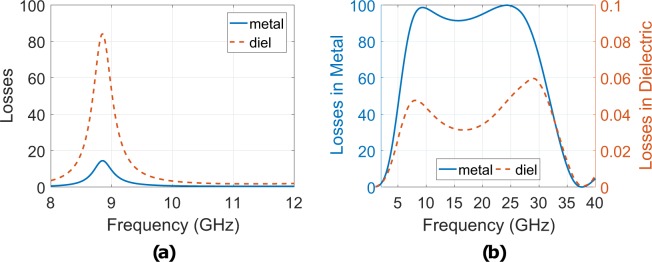
The simulated losses in the copper (**a**) and the printed (**b**) absorber: in the first case the captured energy mainly dissipates in the dielectric parts and the bandwidth is narrow (FBW = 2.1%), while in the second case the bandwidth is much higher (FBW = 125%) but now the captured energy is located in the metallic (resistively loaded) parts (left axis) rather than on dielectric medium (right axis) of the absorber.

Figure 5 presents in which medium (i.e., metallic (metal) or dielectric (diel)) the captured by the absorber energy dissipates. Specifically, it depicts the ratio to percentage of either the *P*_*d*_ or *P*_*m*_ to the total captured power for normal incidence for the copper (Fig. 5a) and the resistively loaded (Fig. 5b) absorber. Based on Fig. 5a it is evident that for the copper absorber the captured energy mainly dissipates in the dielectric parts (above 80%) rather than in the metallic parts (less than 20%). The situation is reversed for the resistively loaded absorber (Fig. 5b): now the losses in the metallic parts dominate (90% to 100%), while the losses in the dielectrics are limited to 0.06%.

**Figure 6:**
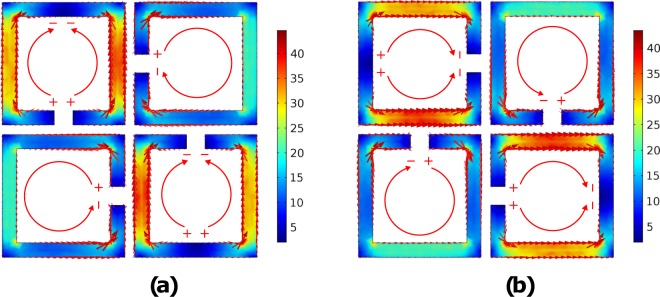
The simulated current distribution for the resistively loaded absorber for both polarisations, i.e., TE (**a**) and TM (**b**) at 9.7 GHz, where the first maxima of absorption occurs (Fig. 4b): now the loops are acting as distributed resistors where the captured energy is transformed to ohmic losses. The dominant loops for each polarisation are those in which their wide parallel arms are in alignment to the electrical field of the incident plane wave.

The surface current distribution for the resistively loaded absorber for normal incidence and for both polarisations, TE or TE is depicted in Fig. 6. Again, the tangential electrical field of the incident plane wave is coupled with the SSRRs and forms surface currents: in the SSRRs, which are symmetrical to the electric component, reverse currents are observed, while in the other SSRRs the currents form loops. However, the mechanism of absorption has now been changed, compared to the copper absorber (Fig. 1g,h): the resistive traces now act as distributed resistors, which transform the captured energy to ohmic losses, and hence, absorbance occurs. All the SSRRs contribute to this phenomenon, but with different weights. Specifically, for the TE-case (Fig. 6a) the arms of the upper-left and down-right SSRR act as distributed resistors, where the captured energy is transformed to ohmic losses, while, the upper-right and the bottom-left SSRRs also contribute to the absorption mechanism as distributed resistors, but with lower impact, since the magnitude of the current in those loops is less. For the TM-case (Fig. 6b) the phenomenon is similar, as expected due to 90-deg. rotational symmetry. The maximum magnitude of the surface currents (≈40 A/m) is now much lower than the copper absorber (≈2.5 × 10^3^ A/m, Fig. 1g,h). The latter occurs due to the fact that the SSRRs now act as distributed resistors and not as resonating elements (copper absorber): the captured energy now is transformed to ohmic losses in the resistively loaded traces and not because of vicinity of strong surface currents to the lossy substrate (copper absorber).

**Figure 7:**
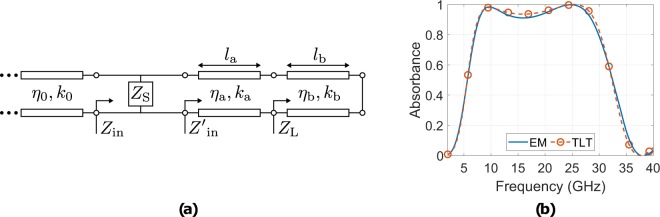
The equivalent circuit schematic for the resistively loaded absorber (**a**) it is assumed that *Z*_S_ represents a series RLC circuit, and thus *Z*_S_ = *R* + *jωL* + (*jωC*)^−1^, with *R* = 240.9315 Ω, *L* = 1.7637 nH and *C* = 66.9497 fF. It is also depicted the absorbance as it was estimated through full electromagnetic (EM) and TLT analysis (**b**): good agreement is observed, and thus the assumption of the series RLC circuit is valid.

Next, the proposed geometry was modelled in terms of absorbance using a transmission line equivalent circuit model. The equivalent circuit schematic for the absorber is depicted in Fig. 7. Based again on the TLT and since the transmission line ends to a short-circuit (grounded absorber) is

9 $${Z}_{{\rm{L}}}=j{\eta }_{{\rm{b}}}\,\tan ({k}_{{\rm{b}}}{l}_{{\rm{b}}}),$$

where, *η*_b_, *k*_b_ and *l*_b_ is the characteristic impedance, the wavenumber and the thickness of the foam-substrate. Then,

10 $${Z^{\prime} }_{{\rm{in}}}={\eta }_{{\rm{a}}}\frac{{Z}_{{\rm{L}}}+j{\eta }_{{\rm{a}}}\,\tan ({k}_{{\rm{a}}}{l}_{{\rm{a}}})}{{\eta }_{{\rm{a}}}+j{Z}_{{\rm{L}}}\,\tan ({k}_{{\rm{a}}}{l}_{{\rm{a}}})},$$

where similarly, *η*_a_, *k*_a_ and *l*_a_ is the characteristic impedance, the wavenumber and the thickness of the PET-substrate. Hence, the input impedance of the absorber is given by,

11 $${Z}_{{\rm{in}}}=\frac{{Z}_{{\rm{S}}}{Z^{\prime} }_{{\rm{in}}}}{{Z}_{{\rm{S}}}+{Z^{\prime} }_{{\rm{in}}}},$$

where *Z*_S_ is the equivalent impedance of the resistively loaded pattern (i.e., SSRR loops), and thus, the reflection coefficient is given by,

12 $${\rm{\Gamma }}=\frac{{Z}_{{\rm{in}}}-{\eta }_{0}}{{Z}_{{\rm{in}}}+{\eta }_{0}},$$

and finally the absorbance is estimated through (2) as a function of *Z*_S_. It is noted that in an isotropic medium the characteristic impedance is given by,

13 $$\eta ={\eta }_{0}\sqrt{\frac{{\dot{\mu }}_{r}}{{\dot{\varepsilon }}_{r}}},$$

where, ${\dot{\varepsilon }}_{r}={\varepsilon }_{r}(1-j\,\tan \,{\delta }_{e})$, ${\dot{\mu }}_{r}={\mu }_{r}(1-j\,\tan \,{\delta }_{m})$ is the complex relative permittivity and permeability of a material with electrical ($\tan \,{\delta }_{e}$) and magnetic ($\tan \,{\delta }_{m}$) losses: e.g., in our case for the PET and the foam absorber is ${\dot{\varepsilon }}_{r}=2.5(1-j0.0009)$ and ${\dot{\varepsilon }}_{r}=1.05$, respectively, while for all cases ${\dot{\mu }}_{r}=1$. Additionally, the wavenumber is given by,

14 $$k=\frac{2\pi }{{\lambda }_{0}}\sqrt{{\dot{\varepsilon }}_{r}{\dot{\mu }}_{r}},$$

where *λ*_0_ is the wavelength in the free space.

For the resistively loaded absorber it is assumed that *Z*_S_ is a series RLC circuit, and not a resistance as in the Salisbury screen, and thus, is,

15 $${Z}_{{\rm{S}}}=R+j\omega L+\frac{1}{j\omega C},$$

where, *R*, *L*, *C* is the resistance, inductance and capacitance of the impedance and *ω* is the angular frequency. Based on curve fitting optimization analysis and on the absorbance, which has been estimated though full electromagnetic simulation (Fig. 4b) is *R* = 240.9315 Ω, *L* = 1.7637 nH and *C* = 66.9497 fF. Figure 7b depicts the resistively loaded absorbance versus frequency as estimated through full electromagnetic (EM) and TLT analysis: good agreement is observed, and thus the assumption that *Z*_S_ could be considered as a series RLC circuit appears correct.

**Figure 8:**
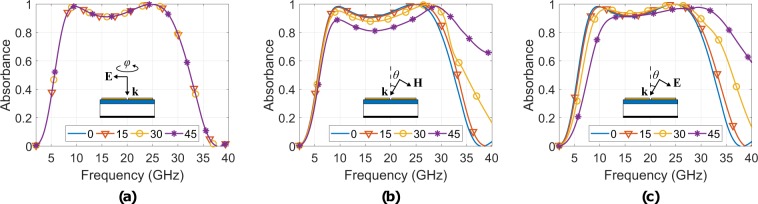
Resistively loaded structure’s simulated absorbance for normal incidence when the electric field **E** rotates by angle *ϕ* from 0 deg. to 45 deg. around the normal to the unit-cell wavevector **k** (**a**), and for oblique incidence for both polarizations TE (**b**) and TM (**c**) when the angle of incidence *θ* varies from 0 deg. to 45 deg.

Figure 8a depicts the simulated resistively loaded structure’s absorbance for normal incidence as the electric (or magnetic, as well) field component of the incident plane wave steers from 0 to 45 deg. (again, angles greater than 45 deg. were not tested since the super-cell presents 45 deg. symmetry): it is evident that the absorber is polarisation insensitive for normal incidence. The latter occurs because of the 90 deg. super-cell symmetry.

Next, the polarization insensitivity was simulated, but now for oblique incidence. The TE-case is depicted in Fig. 8b. As angle of incidence *θ* varies from 0 to 45 deg., the absorbance remains higher than 0.8 from 7.7 to 29.9 GHz, resulting FBW = 118%, but is changing shape: a normal magnetic field component to the SSRRs arises as *θ* increases, which induces surface currents, which in turn contribute to the absorption mechanism constructively or destructively: the *A* decrement is because tangential electric field currents and the normal magnetic field currents operate destructively, and hence, the latter is most noticeable for higher *θ*. However, the major contribution to the absorbance is because of the tangential electric field, which remains constant as *θ* increasing. Thus, the *A* is changing shape but is not significantly shifting in frequency. Additionally, due to the wideband operation, there are no distinct resonance defined maxima, as there were for the copper-absorber.

Figure 8c presents the absorbance for oblique incidence and for TM polarization. As angle *θ* increases from 0 to 45 deg., *A* remains higher than 0.8 from 9.9 to 29.9 GHz, resulting FBW = 100.5%. Now, the tangential electric component is decreasing as the angle of incidence increases. Thus, this induces through the electric field surface currents form equivalently smaller electrical traces and loops, and hence, a shift of the bandwidth to higher frequencies is observed.

### Bent Printed Absorber

**Figure 9:**
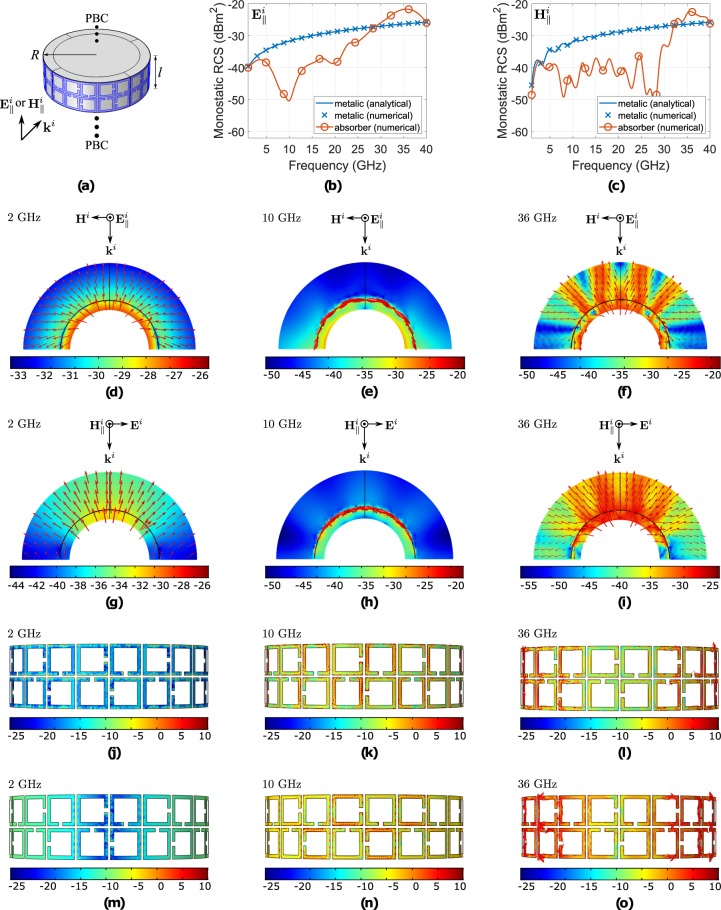
A section (ring) of the infinite cylinder (**a**), which is covered by the bent printed absorber. The simulated monostatic RCS versus frequency of the latter in contrast to the RCS of a metallic cylinder with exactly the same radius, for normal incidence and for ${{\bf{E}}}_{\parallel }^{i}$ (**b**) or ${{\bf{H}}}_{\parallel }^{i}$ (**c**) polarization. Also, the scattered electric field (half cylinder, top-view) (**d**–**i**) and the induced surface currents distribution in the SSRRs (side-view) (**j**–**o**) for various frequencies and for both polarization cases.

The resistively loaded absorber was curved and its absorbance was tested. In particular, a metallic circular cylinder with infinite height was covered by the absorber (Fig. 9a) and the monostatic radar cross section (RCS) versus frequency of the latter geometry (i.e., *σ*_*a*_) was estimated through full electromagnetic analysis when the incident electrical ${{\bf{E}}}_{\parallel }^{i}$ or magnetic ${{\bf{H}}}_{\parallel }^{i}$ field is in parallel to the cylinder: 10 super-cells covered its periphery, and thus, the latter has radius of *R* = 5*l*/*π*, where *l* = 12.59 mm is the super-cell’s size, as mentioned before. The analytical and simulated (numerical) monostatic RCS of a metallic cylinder (i.e., *σ*_*m*_) with exactly the same radius was estimated, also, for comparison. Based on the literature^46^, the two dimensional RCS (i.e., *σ*^2D^) of a metallic circular cylinder of radius *R* and of infinite height, when a plane wave of ${{\bf{E}}}_{\parallel }^{i}$-polarisation normally impinges it, is given by^46^

16 $${\sigma }^{2{\rm{D}},{{\bf{E}}}_{\parallel }^{i}}=\mathop{\mathrm{lim}}\limits_{\rho \to \infty }[2\pi \rho \frac{{|{{\bf{E}}}_{\parallel }^{s}|}^{2}}{{|{{\bf{E}}}_{\parallel }^{i}|}^{2}}]=\frac{4}{k}{|\mathop{\sum }\limits_{n=-\infty }^{+\infty }\frac{{J}_{n}(kR)}{{H}_{n}^{(2)}(kR)}{e}^{jn\varphi }|}^{2},$$

where *J*_*n*_ is Bessel function of the first kind, ${H}_{n}^{(2)}$ is Bessel function of third kind (Hankel function), *k* the wavenumber, *R* the radius of the cylinder, *ϕ* is the azimuthal angle and {^*i*^}, {^*s*^}, denotes the incident plane wave and the scattered by the cylinder electric field. In our case, where a ring of hight *l* is periodically repeated (Fig. 9a), the three dimensional RCS is given by^46^,

17 $${\sigma }^{3{\rm{D}},{{\bf{E}}}_{\parallel }^{{\rm{i}}}}\underline{\approx }\frac{4{l}^{2}}{\pi }{|\mathop{\sum }\limits_{n=-\infty }^{+\infty }\frac{{J}_{n}(kR)}{{H}_{n}^{(2)}(kR)}{e}^{jn\varphi }|}^{2}=\frac{k{l}^{2}}{\pi }{\sigma }^{2{\rm{D}},{{\bf{E}}}_{\parallel }^{i}}.$$

Similarly, for the ${{\bf{H}}}_{\parallel }^{i}$-polarisation is^46^

18 $${\sigma }^{2{\rm{D}},{{\bf{H}}}_{\parallel }^{i}}=\mathop{\mathrm{lim}}\limits_{\rho \to \infty }[2\pi \rho \frac{{|{{\bf{E}}}_{\parallel }^{s}|}^{2}}{{|{{\bf{E}}}_{\parallel }^{i}|}^{2}}]=\frac{4}{k}{|\mathop{\sum }\limits_{n=-\infty }^{+\infty }\frac{{J^{\prime} }_{n}(kR)}{{H}_{n}^{(2)^{\prime} }(kR)}{e}^{jn\varphi }|}^{2}$$

and^46^

19 $${\sigma }^{3{\rm{D}},{{\bf{H}}}_{\parallel }^{i}}\underline{\approx }\frac{4{l}^{2}}{\pi }{|\mathop{\sum }\limits_{n=-\infty }^{+\infty }\frac{{J^{\prime} }_{n}(kR)}{{H}_{n}^{(2)}(kR)}{e}^{jn\varphi }|}^{2}=\frac{k{l}^{2}}{\pi }{\sigma }^{2{\rm{D}},{{\bf{H}}}_{\parallel }^{i}},$$

where {′} denotes the partial derivative with respect to the entire argument of the Bessel functions The results for the monostatic RCS are depicted in Fig. 9b,c. There are three distinct regions: first when *σ*_*a*_ = *σ*_*m*_, where the absorber is *outside the operating frequency range*, the plane wave penetrates it and finally is reflected by the absorber’s ground (i.e., metallic circular cylinder of radius *R* − *h*_1_ − *h*_2_), second when *σ*_*a*_ < *σ*_*m*_, where the absorber is *inside the operating frequency range* and captures the incidence RF power, and third when *σ*_*a*_ > *σ*_*a*_, where the SSRRs become resonant and act as radiating elements in an array configuration which steer the scattered power back, as will explained. For ${{\bf{E}}}_{\parallel }^{i}$-polarization, the cylinder absorbs power from 2.5 GHz to 28.7 GHz while for ${{\bf{H}}}_{\parallel }^{i}$-polarization from 2.9 GHz to 34.1 GHz. In order to shed further light on the operation mechanism, the power flow (time average) of the scattered field (vectors and magnitude in dB) is also depicted for the mentioned three regions (at 2, 10 and 36 GHz) and for both polarizations (${{\bf{E}}}_{\parallel }^{i}$ and ${{\bf{H}}}_{\parallel }^{i}$-polarization). At 2 GHz (Fig. 9d,g), the plane wave propagates inside the absorber until it is uniformly reflected by geometry’s ground. At 10 GHz (Fig. 9e,h), it is observed that the scattered energy mainly dissipates in the SSRRs, it is not reflected back, and thus, it is absorbed. At 36 GHz (Fig. 9f,i), it is observed that the incident wave is not scattered by the ground, as at 2 GHz, but mainly via specific SSRRs, which reflect the reflected energy to the exactly position of the incident wave. Figure 9j–l and Fig. 9m–o depicts the surface current distribution in the SSRRs for ${{\bf{E}}}_{\parallel }^{i}$ and ${{\bf{H}}}_{\parallel }^{i}$-polarization, respectively, at 2, 10 and 36 GHz. At 2 GHz, it is evident that the surface current distribution in the loops is very low, as expected. At 10 GHz, the SSRRs interact with the incident wave, and strong surface currents appear in all the loops, as has been explained previously, and the absorption mechanism is activated. At 36 GHz the incident wave forms again surface currents in the loops, mainly at the side of the cylinder, the mechanism changes and the geometry is not working as absorber. Specifically, the strong surface currents in the SSRRs are in distance of *h*_1_ + *h*_2_ = 3.89 mm from the ground, and thus, they resonate in distance ~0.47*λ*_36 GHz_ from a metallic cylinder of radius *R* − *h*_1_ − *h*_2_, and hence, form an array which steers the energy back. The latter is the reason that the *σ*_*a*_ at that frequency is higher than the *σ*_*m*_ of a solid metallic cylinder of radius *R*.

Next, the captured power by the metallic and dielectric parts of the bent absorber was estimated through (8) and (7), respectively. The time averaged incident power to the curved absorber is found to be

20 $${P}_{in}=\mathop{\iint }\limits_{{S}_{m}}\,{{\bf{P}}}^{i}\cdot {\bf{dS}}=\mathop{\iint }\limits_{{S}_{m}}\,\frac{{|{{\bf{E}}}^{i}|}^{2}}{2{\eta }_{0}}\hat{{\bf{n}}}\cdot {\bf{dS}},$$

evaluated over the *S*_*m*_ surface of a cylinder with radius *r*, which encapsulates the bent absorber, where **dS** is the unit normal vector of latter surface, $\hat{{\bf{n}}}={{\bf{k}}}^{i}/|{{\bf{k}}}^{i}|$ is the unit vector which denotes the propagation direction and **P**^*i*^ is the time average of the power flow of the incident plane wave. Assuming that |**E**^*i*^| = 1 V/m, the bent absorber lies on a cylinder with *z*-axis and the incident plane wave propagates to *x*-axis is found to be

21 $${P}_{in}=\frac{Rl}{{\eta }_{0}}.$$

It is noted that, only the sub-area where *x* < 0 was taken into account in our calculations, i.e., the part which is facing the incident plane wave.

**Figure 10:**
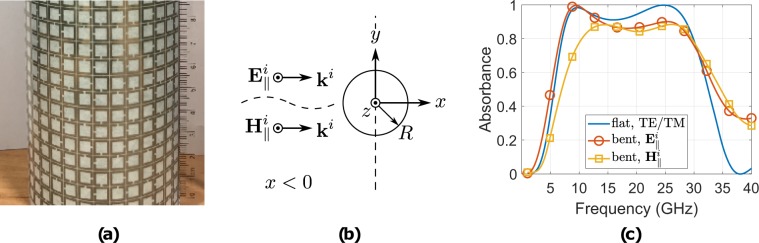
The bent printed absorber (**a**), the cross-section of the cylinder (**b**), which was surrounded by the latter and the simulated absorbance of the bent printed absorber for both polarizations.

Next, the ratio of the power dissipated in the metallic and dielectric parts of the bent absorber to the incident power (time average) it is defined,

22 $$A^{\prime} =\frac{{P}_{m}+{P}_{d}}{{P}_{in}},$$

which is a metric of the amount of the power which is captured by the absorber, i.e., equivalent metric for the absorbance but for the bend version: Fig. 10 depicts the *A*′ and *A* of the flat resistively loaded absorber for normal incident: for the ${{\bf{E}}}_{\parallel }^{i}$ and ${{\bf{H}}}_{\parallel }^{i}$-polarisation, *A*′ remains higher than 0.8 for 6.6–29 GHz and for 10.5–29.6 GHz, respectively, resulting FBW = 126% and FBW = 95%, respectively. Moreover, for higher frequencies, greater than 30 GHz, the equivalent absorbance for the bent geometry remains higher than 0.3, which is in alignment with the observation that at these frequencies strong surface currents appear in the SSRRs, making the latter act as resonators, which steer the scattered energy back.

Based on the above analysis, the proposed resistively loaded absorber presents high performance in terms of absorbance either when deployed as flat or curved, is wideband with FBW higher than 125% for normal incidence, polarisation insensitive and wide-angle, up to 45 deg.

## Methods

### Simulation setup

All the presented absorbers were simulated in terms of their absorbance via COMSOL Multiphysics^40^ with finite element method. For the flat periodic absorbers, periodic boundary conditions (PBC) were applied around the super-cell, which was illuminating by a plane wave (*periodic port*), which in tern, normally or by an angle impinges to the structure with a specific polarization. On the bottom of the supper-cell perfect electric condition (PEC) was applied, while in the top perfect matched layer (PML) condition. The absorbance *A* was estimated here through the reflection coefficient parameter. The printed, resistively loaded traces were simulated as ohmic sheets of zero thickness and surface resistance of 27 Ω/sq. For the bent printed absorber, only the ring presented in Fig. 9a was simulated and the RCS was estimated: PBC were applied on the top and bottom of the ring, PML was around the ring, while the structure was solved for the scattered field. The absorbance *A*′ was estimated here through (22), as has been explained.

### Fabrication Process

**Figure 11:**
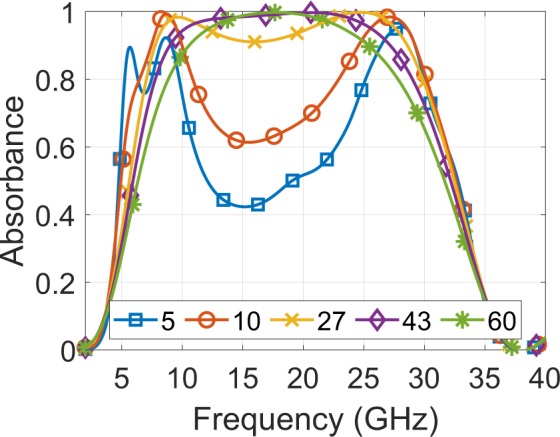
Simulated absorbance for normal incidence versus frequency for various values of the surface resistance (5,10,27,43 and 60 Ω/sq).

For the fabrication of the printed absorber the resistively loaded traces were printed on a 0.14 mm thick, polyethylene terephthalate (PET) sheet (*ε*r = 2.5, losses of tan*δ* = 0.0009) by using the conventional, unmodified inkjet printer Epson Stylus C88+. The printer deposited ink consisting of electrically conductive ink MetalonTM JS-B25P^45^, consisting of silver nanoparticles (Ag content 25 wt%), diluted with water as the solvent. During the fabrication process, three factors determine the achieved surface resistance. First is the ratio of ink and solvent, the second is the RGB-code used during the printing process and finally the curing process after the printing (i.e. the post processing step) for the evaporation of the solvent. Thus, in order to achieve the desired surface resistance (i.e., 27 Ω/sq) first, a mixture of 1-part ink to 5-part solvent (i.e., 1:5 volume ink-solvent ratio) and second, RGB-code of (29,29,29) was utilised based on^39^ results. Any change on the mixture or RGB-code will modify the resulting surface resistance. In general, a curing process in an electric oven is often used evaporate any solution in the ink and bond the silver nanoparticles together, leading to a decrease in surface resistance, hence this technique is usually used for printing low resistively loaded structures. In our work, where the surface resistance is relativity high (i.e., 27 Ω/sq) for the specified ink-solvent mixture and RGB-code, we do not “bake” the printed surface, but instead we leave it to slowly evaporate the solvent at room temperature (48 hours at 20°C), thereby allowing fewer nano particles to bond and achieving a higher surface resistance. Based on^39^, where no oven was used after printing, the measured surface resistance for 1:5 volume ink-solvent ratio and for RGB-code (25,25,25), (27,27,27) and (30,30,30) is 5 Ω/sq, 10 Ω/sq and 43 Ω/sq, respectively. Thus, applying curve fitted in our work we have chosen RBG-code of (29,29,29) to achieve a surface resistance of 27 Ω/sq. Additionally, based on^39^, it is evident that the RGB-code controls the spatial density of the droplets dispensed by the printer: RGB-code (0,0,0) is black (maximum spatial density of the ink, hence minimum surface resistance) and RGB-code (255,255,255) is white (no ink, hence maximum surface resistance) with RGB values in between (i.e., grayscale) resulting in resistivity control between the available maximum and minimum values. Figure 11 depicts the resulting absorbance versus frequency for various levels of surface resistance: change on surface resistance from 5 Ω/sq to 60 Ω/sq modifies the resulting absorbance, however from 27 Ω/sq to 60 Ω/sq the change is marginal and affects mainly to the absorbance bandwidth. Thus, the proposed structure is robust in terms of surface resistance fluctuations during fabrication.

### Measurement setup

**Figure 12:**
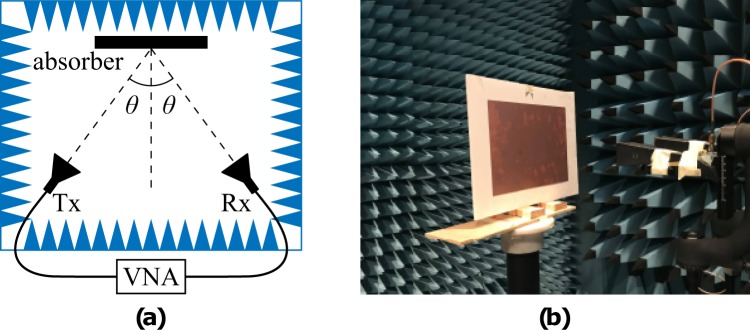
Experimental setup (**a**) of the measurements (**b**).

The measurement setup is depicted in Fig. 12. A two-ports vector network analyser was connected to the receiver (Rx) and transmitter (Tx) horn antennas, while at far-field distance, was placed the electromagnetic absorber. For normal incidence, Rx-Tx were collocated in front of the absorber, while for oblique incidence of angle *θ*, the horn antennas were moved in distance 2*θ*. For each combination (i.e., type of absorber, polarization, angle of incidence) the transmission coefficient *S*_21_ was measured for three cases: first for the absorber ${S}_{21}^{\,a}$, second for a metallic plate of exactly the same dimensions to the absorber ${S}_{21}^{\,m}$, and third, for the empty space (i.e., the absorber was removed) ${S}_{21}^{\,n}$. The absorbance was estimated as ${S}_{21}^{\,m}-{S}_{21}^{\,a}-{S}_{21}^{\,n}$, i.e., *differential measurement*. Time domain gating and smoothing techniques were applied over the measured data. All the above measurements took place inside anechoic chamber. In order to verify the exact thickness of the printed resistively loaded traces, measurement was performed through the stylus-based method and specifically by using the Tencor Stylus profilometer: the measured thickness was found to vary from 0.2 to 0.4 *μ*m. Figure 4c depicts the measured, printed, resistively loaded traces through a microscope (10× zooming). It is noted that, since the resistively loaded traces have been simulated as ohmic sheets with surface resistance of 27 Ω/sq, the measured thickness was not taken into account in our calculations.

## Conclusion

This work presented the first flexible, polarization insensitive, wide-angle, ultra wideband, electrically thin electromagnetic absorber, which was easily fabricated by using a conventional inkjet printer by utilizing conductive ink, which diluted in water, stored in an empty inkjet cartridge and used by the printer. The design of the absorber is based on a versatile and systematic technique, which was presented and tested in this work: the basic idea is the form of super-cells with 90 degrees rotational symmetry. The presented absorber was tested in terms of absorbance through numerical analysis and measurements, where a very good agreement was observed between the simulated and measured results. This novel electromagnetic absorber is a perfect candidate for stealth technology applications, since it is able to cover not only flat surfaces, but also curved objects, operating for extended FBW, higher than 125%, regardless polarization, up to 45 deg. angle of incidence.

To the best of the authors knowledge and based on the literature, the proposed structure is the first flexible, ultra-wideband, polarization insensitive, wide-angle, easily fabricated through conventional inkjet printing techniques, electrically thin electromagnetic absorber.
